# Patterns of maternal adverse childhood experiences and the intergenerational association of preschool children’s emotional and behavioral problems

**DOI:** 10.3389/fpsyt.2024.1431475

**Published:** 2024-08-13

**Authors:** Ruoyu Li, Wan Xiao, Jun Wu, Yang Zhou, Jinhong Zha, Danni Wang, Tian Xing, Yuhui Wan

**Affiliations:** ^1^ Department of Maternal, Child & Adolescent Health, School of Public Health, Anhui Medical University, Hefei, Anhui, China; ^2^ Teaching Center for Preventive Medicine, School of Public Health, Anhui Medical University, Hefei, Anhui, China; ^3^ Key Laboratory of Oral Disease Research of Anhui Province, Stomatologic Hospital and College, Anhui Medical University, Hefei, China

**Keywords:** adverse childhood experiences, emotional and behavioral problems, latent class analysis, gender differences, preschool children

## Abstract

**Introduction:**

Children of whose mothers exposed to adverse childhood experiences (ACEs) are at increased risk for developmental problems. This study aims to investigate the relationship between types and patterns of maternal ACEs and preschool children’s emotional and behavioral problems (EBPs) in China, and to explore gender differences associated with these problems.

**Methods:**

In this cross-sectional study, we selected 9,647 children from 36 preschools in three cities of Anhui province, China. Questionnaires were used to report the details of maternal ACEs and children’s EBPs. We used the latent class analysis (LCA) to identify “patterns” in the types of maternal ACEs. Binomial logistic regressions was performed to examine the relationship between types and patterns of maternal ACEs and preschoolers’ EBPs.

**Results:**

Latent class analysis (LCA) revealed four different classes of maternal ACEs. Logistic regression analysis showed that compared with the low ACEs class, children of mothers in the high abuse and neglect class had the highest risk of developing EBPs (*OR* = 5.93, 95%*CI*: 4.70-7.49), followed by moderate ACEs class (*OR* = 2.44, 95%*CI*: 1.98-3.00), and high household dysfunction class (*OR* = 2.16, 95%*CI*: 1.19-3.90). We found gender differences in the effects of high abuse and neglect/moderate ACEs class and maternal childhood physical abuse/neglect on children’s EBPs, which had a stronger impact on EBPs in boys than girls (*P*<0.05).

**Discussion:**

This study supports and refines existing research that confirms an intergenerational association between types and patterns of maternal ACEs and children’s EBPs in a large Chinese sample, so as to provide references for the early prevention and control of children’s EBPs.

## Introduction

Emotional and behavioral problems (EBPs) include a series of emotional problems and behavioral problems, such as anxiety or depression symptoms, aggressive behavior, etc (1). Studies showed that EBPs are common among preschoolers in many countries, including China (2–4). Preschool age is a crucial period for the development of children’s emotional and social maladjustment. During this period, children are susceptible to interference from various internal and external environmental factors, leading to a series of EBPs (5). EBPs in early childhood tend to continue into later life, such as mid-childhood, adolescence, and adulthood, with profound and long-term impacts on individuals, families, and society (6, 7). Evidence from previous researches suggests that children’s EBPs are associated with family stressors, including low socioeconomic status (8), tense family relationships (9), mental health problems of parents (10), and maternal ACEs (11, 12).

ACEs were defined as childhood abuse, neglect, peer bullying, community and collective violence, and household dysfunction before the age of 18 (13). As reported by the World Health Organization (WHO), over one-third of the global population has been exposed to at least one ACE (14). Previous research had demonstrated a consistent association between childhood adversity and a range of adverse outcomes, including risky health behaviors (15), mental health problems (16, 17), and common chronic diseases (18, 19). These findings highlight the significance of early screening for ACEs. In order to effectively assess ACEs, the WHO has developed the Adverse Childhood Experiences International Questionnaire (ACE-IQ), which comprehensively measures individuals’ ACEs before the age of 18 from a multi-dimensional perspective (20). The questionnaire has been validated in samples from a multitude of countries worldwide. For example, a national survey of 10,156 adults in Saudi Arabia indicated that 80% of participants had experienced at least one ACE (21). A Korean study revealed that approximately 50% of the surveyed college students had experienced at least one ACE (22). A Chinese study revealed that 75% of the surveyed adults had experienced at least one ACE, while 19% reported four or more ACEs (23).

A growing body of research suggests that parental ACEs contribute to adverse health outcomes in offspring (8, 24) and increase the risk of emotional (25, 26) and behavioral (27, 28) problems in children. For instance, Kumar et al. (29) found that maternal ACEs may increase the risk of internalizing and externalizing problems in children. In the context of Chinese culture, mothers exert a significant influence on children’s healthy development. Consequently, as research in this field has progressed, domestic scholars have also focused on the intergenerational health effects of maternal ACEs. Zhu et al. (30) conducted an investigation of 2282 mother-child dyads in China, finding that maternal ACEs were positively related to children’s behavioral problems. A cross-sectional study demonstrated that maternal ACEs may increase the risk of EBPs in offspring (31). The above studies suggest that maternal ACEs are important factors affecting the healthy development of preschool children. However, there is a paucity of research on intergenerational health effects in China, and further investigation is required to elucidate the relationship between maternal ACEs and preschool children’s EBPs.

Previous research on the assessment of ACE have concentrated on single types of exposure rather than combined exposure patterns (32). A study showed that ACEs are prevalent in China, with over 60% of adults in the country having experienced at least one ACE (33). The Kaiser Permanente ACE study also indicated that a single ACE experience was a significant predictor of at least one additional ACE experience (34). In light of the high prevalence of multiple ACEs types in real-life settings, a single type estimate may not fully capture the complexity of ACE exposure in daily life. It is therefore necessary to evaluate the combined exposure pattern of ACEs. It is well documented that there are a number of methods for evaluating the combined ACEs. These include the cumulative risk model (35), the dimension model of adversity (which categorizes ACEs into two dimensions: deprivation and threat; deprivation mainly includes neglect experiences, while threat includes abuse and community/family violence experiences) (36), and the latent class analysis (LCA) (37). Some scholars have highlighted the superiority of the LCA in addressing ACEs of different dimensions and in analyzing retrospective data (38, 39). Furthermore, several studies have demonstrated that various types of ACEs can result in disparate health outcomes. For example, Wang et al. (40) observed that different types of maternal ACEs were associated with behavioral problems in preschoolers, with children whose mothers had experienced peer bullying and emotional abuse exhibiting a higher risk of developing behavioral problems. These findings underscore the necessity for a comprehensive examination of the combined exposure pattern of maternal ACEs and an investigation into the intergenerational health effects of multiple maternal ACEs on children.

In addition, we found gender differences in the association between ACEs of mothers and EBPs in preschool children. A longitudinal study indicated a important pathway from persistent ratings of maternal childhood sexual abuse severity to an increase in externalizing behavioral problems from ages 4 to 8 in offspring, the pathway was significant in the mother–daughter dyads but not significant in the mother–son dyads (41). Oshio et al. (42) found that compared with the association between maternal childhood abuse and sons’ behavioral problems, daughter’ behavioral problems were more likely associated with maternal childhood abuse history. Therefore, gender differences in maternal ACEs and offspring’s EBPs need to be further explored.

The purpose of the current study is to analyze the exposure patterns of maternal ACEs using the LCA model, and to examine the intergenerational association between different types and patterns of maternal ACEs and the EBPs in preschool children. Subsequently, it explores the possible gender difference in this association.

## Materials and methods

### Sample and procedure

This cross-sectional study was conducted in Fuyang, Wuhu, and Lu’an, in China. A multistage cluster sampling method was conducted to select three cities that are broadly representative of the average population within Anhui province in terms of economic development and demographic composition. Six urban and six rural kindergartens were randomly selected in each city and all children aged 3-6 in these kindergartens were recruited as the research subjects. Inclusion criteria: (1) The children have no mental or physical diseases; (2)The mothers were willing to participate in the survey after informed consent. (3)The children are between 3-6 years old. Mothers were asked to fill out an anonymous questionnaire through the “Wenjuanxing”. Of the preschool children recruited, 321 responses filled out by other caregivers were excluded from the sample, and 30 children younger than 3 years and older than 6 years were excluded. Thus, a total sample of 9,647 (96.5%) participants were finally chosen in the survey from May to June 2021 (**Supplementary Figure S1**). The study design and data collection procedures were both approved by the Ethics Committee of Anhui Medical University (20210655).

### Measures

#### Adverse childhood experiences

Maternal ACEs were measured by Adverse Childhood Experiences International Questionnaire (ACE-IQ) (20), which has demonstrated good reliability and validity in a Chinese context (23). The questionnaire includes the following categories: emotional abuse (two items); physical abuse (two items); sexual abuse (two items); emotional neglect (two items); physical neglect (three items); peer bullying (three items); community violence (two items); household dysfunction (including five items: “Did your parents or guardian fight with each other?” “Did you live with a household member who was a problem drinker or alcoholic, or misused street or prescription drugs?” “Did you live with a household member who was depressed, mentally ill, or suicidal?” “Were your parents ever separated or divorced?” “Did your mother, father, or guardian die?”). Except for household dysfunction, each item was rated on a 5-point Likert scale (0 = never, 1= occasionally, 2 = sometimes, 3 = often, 4 = always). Using the binary scoring, in each category, if the mother answered “never”, it was coded as “0”, and if the answer was “occasionally,” “sometimes,” “often,” or “always,” it was coded as “1”. In household dysfunction, each item adopted a 2-point Likert scale, ranging from 0 (No) to 1 (Yes). Each item of household dysfunction was treated as a category. Ultimately, twelve categories of ACEs were included in the analysis. The scores for each category were converted into binary categorical variables, which were then incorporated into the LCA model.

#### Emotional and behavioral problems

The Strength and Difficulties Questionnaire (SDQ) was developed by Goodman (43) to evaluate children’s EBPs, and it is suitable for the assessment and screening of EBPs among children and adolescents aged 3–16 years (44). This scale has good reliability and validity in Chinese preschool children (4). The SDQ is a 25-item measure, divided into five domains: conduct problems, hyperactivity/inattention, emotional symptoms, peer problems, and prosocial problems with peers (45) (**Supplementary Table S1**). Each item was evaluated on a 3-point scale (0 = “not true”, 1 = “somewhat true”, 2 = “certainly true”). We used the total difficulty score to determine whether the tested child has EBPs. The total difficulty recommended cut-off points are: 0–13 points to categorize normal scores, 14–16 for borderline scores, and 17–40 for abnormal scores (46). Consistent with previous research, a dichotomous variable was created for each SDQ subscale in this study, comparing scores classified as “normal/borderline” with scores classified as “abnormal” (47). Given that there are two different evaluation criteria for the SDQ, we evaluated the effect of grouping the “borderline” scores together with the “abnormal” scores (i.e., modelling “normal” versus “borderline/abnormal”) in sensitivity analyses (48). Cronbach’s alpha for the SDQ was 0.70 in this study.

### Covariates

In consideration of prior studies on the confounding factors (31, 40), and the results of univariate analysis (**Table 1**), the covariates in this analysis included residency (Fuyang, Wuhu, Lu’an), child gender (boys, girls), birth weight (<2.5 kg, 2.5-4 kg, ≥4 kg), maternal age, premature birth (no, yes), maternal education level (less than junior high school, high school or technical secondary school, junior college or more), family income (≤6000RMB, 6000-10000RMB, >10000RMB), family structure (extended family, core family, other).

**Table 1:** Background and demographic information for overall sample.

Variables		Total	EBPs		*P*-value
			No(N,%)	Yes(N,%)	
Residency	Fuyang	4100(42.5)	3865(94.3)	235(5.7)	<0.001
	Wuhu	3013(31.2)	2841(94.3)	172(5.7)	
	Lu’an	2534(26.3)	2315(91.4)	219(8.6)	
Child gender	Boys	4983(51.7)	4628(92.9)	355(7.1)	0.009
	Girls	4664(48.3)	4393(94.2)	271(5.8)	
Premature birth	No	9164(95.0)	8587(93.7)	577(6.3)	0.001
	Yes	483(5.0)	434(89.9)	49(10.1)	
Birth weight	<2.5kg	405(4.2)	359(88.6)	46(11.4)	<0.001
	2.5−4kg	7737(80.2)	7255(93.8)	482(6.2)	
	≥4kg	1505(15.6)	1407(93.5)	98(6.5)	
Only child	Yes	6196(64.2)	5777(93.2)	419(6.8)	0.144
	No	3451(35.8)	3244(94.0)	207(6.0)	
Breastfeeding time	≤6 months	3181(33.0)	2958(93.0)	223(7.0)	0.081
	6 months−1 year	4517(46.8)	4220(93.4)	297(6.6)	
	>1 year	1949(20.2)	1843(94.6)	106(5.4)	
Mother’s education level	Less than junior high school	2197(22.8)	1968(89.6)	229(10.4)	<0.001
	High school or technical secondary school	2619(27.1)	2448(93.5)	171(6.5)	
	Junior college or more	4831(50.1)	4605(95.3)	226(4.7)	
Family income	≤6000RMB	4599(47.7)	4255(92.5)	344(7.5)	<0.001
	6000-10000RMB	3209(33.3)	3012(93.9)	197(6.1)	
	>10000RMB	1839(19.1)	1754(95.4)	85(4.6)	
Family structure	Extended family	4028(41.8)	3735(92.7)	293(7.3)	<0.001
	Core family	5373(55.7)	5070(94.4)	303(5.6)	
	Other	246(2.6)	216(87.8)	30(12.2)	
Number of ACEs type	0	954(9.9)	939(98.4)	15(1.6)	<0.001
	1	2686(27.8)	2592(96.5)	94(3.5)	
	2	2081(21.6)	1976(95.0)	105(5.0)	
	3	1373(14.2)	1285(93.6)	88(6.4)	
	4+	2553(26.5)	2229(87.3)	324(12.7)	

### Statistical analysis

To determine clusters of maternal ACEs, we estimate LCA models to explore mutually exclusive “classes” of 12 maternal ACEs by Mplus 7.4. The maternal ACEs were determined based on best-fitting model indices: Akaike information criterion (AIC), Bayesian information criterion (BIC), adjusted BIC (aBIC), Entropy, Lo-Mendel Rubin Adjusted Likelihood Ratio Test (LMRT-LRT), and Bootstrapped Likelihood-Ratio Test (BLRT) (49–51). Smaller values of AIC and BIC indicate better model fit. Significant LMR-LRT and BLRT values suggest that the k-class model is better than the k-1 class model. The relative entropy value was close to 1.00, manifesting that the tested individuals in the sample were well classified, and average posterior class probabilities greater than 0.70, indicating the individuals in the sample were well classified. After evaluating the optimal fitting model, the modified three-step Mplus procedures (R3STEP auxiliary command) developed by Vermunt (52) was used to determine the relationship between demographic covariates and class membership to make sure that the impacts of the covariates on the classes were minimally biased. The next step involved a series of binary logistic regression models to explore the association between maternal ACEs exposure patterns and offspring’s EBPs, taking into account the influence of child gender, birth weight, only child, maternal age, premature birth, and so on. The study also tested whether the correlations varied by gender through the values of two odds ratios (RORs) (53).

## Result

### Characteristics of participants

**Supplementary Table S2** reports descriptive characteristics for analysis variable, including 9,647 mothers who filled out the questionnaire, the mothers’ mean age was 33.3 years (SD = 4.4); the average number of ACEs experienced by mothers was 2.56 (SD = 2.0); 18.8% of mothers had emotional abuse experience, 22.1% had physical abuse experiences, 3.5% had sexual abuse experiences, 84.3% had emotional neglect experiences, 28.0% had physical neglect experiences, 14.2% had peer bullying experiences, 15.0% had community violence experiences, 36.6% had experiences of parents or guardian fighting, 10.8% had experiences of family members with alcohol or gambling problems, 3.5% had experiences of living with a household member who was depressed, mentally ill, or suicidal, 7.4% had experiences of parental divorce or separation, and 11.5% had experiences of the death of their mother, father, or guardian. 90.1% of the mothers reported exposure to at least one ACE type. 27.8% of mothers reported two types of ACE exposures. The frequencies of maternal exposure to two and three types of ACEs were 21.6% and 14.2%, respectively. 26.5% of mothers had an ACE score of four or more.

As **Table 1** summarizes, including 9,647 preschool children surveyed, the mean age was 5.1 years (SD = 0.9), and 51.7% were boys. A total of 626 preschool children (6.5%) reported EBPs in the past 6 months. Chi-square (*χ* ^2^) tests results showed that there were statistically significant differences in residency, child gender, premature birth, birth weight, mother’s education level, family income, and family structure (*P*<0.05). To prevent the omission of confounding factors that may affect dependent variables, we included candidate variables with *P* values < 0.05 in the univariate analysis, which were eventually included in the binary logistic model.

### Latent class analysis

**Table 2** displayed a 4-class model of maternal ACEs, which showed significant LMR (*P<*0.001) and BLRT (*P<*0.001). Although the AIC and BIC values of the 5-class solution were slightly lower than the 4-class model, the LMR and BLRT results showed that the model was not significantly better than the preceding solutions. Furthermore, the 4-class model has a higher entropy (0.71).

**Table 2:** Indicators of fit for models with one through five latent classes for maternal ACEs.

CLASS	K	AIC	BIC	aBIC	Entropy	LMR	BLRT
1	12	92522.480	92608.573				
2	25	84258.240	84437.600	84358.154	0.75	<0.001	<0.001
3	38	82687.569	82960.196	82839.438	0.84	<0.001	<0.001
4	51	81944.636	82310.531	82148.461	0.71	<0.001	<0.001
5	64	81650.862	82110.023	81906.641	0.68	0.1327	<0.001

ACEs, Adverse childhood experiences; AIC, Akaike’s Information Criteria; BIC, Bayesian Information Criteria; aBIC, sample size adjusted BIC; LMRT, Lo-Mendell-Rubin Test; BLRT, Likelihood Ratio Test.

**Figure 1:**
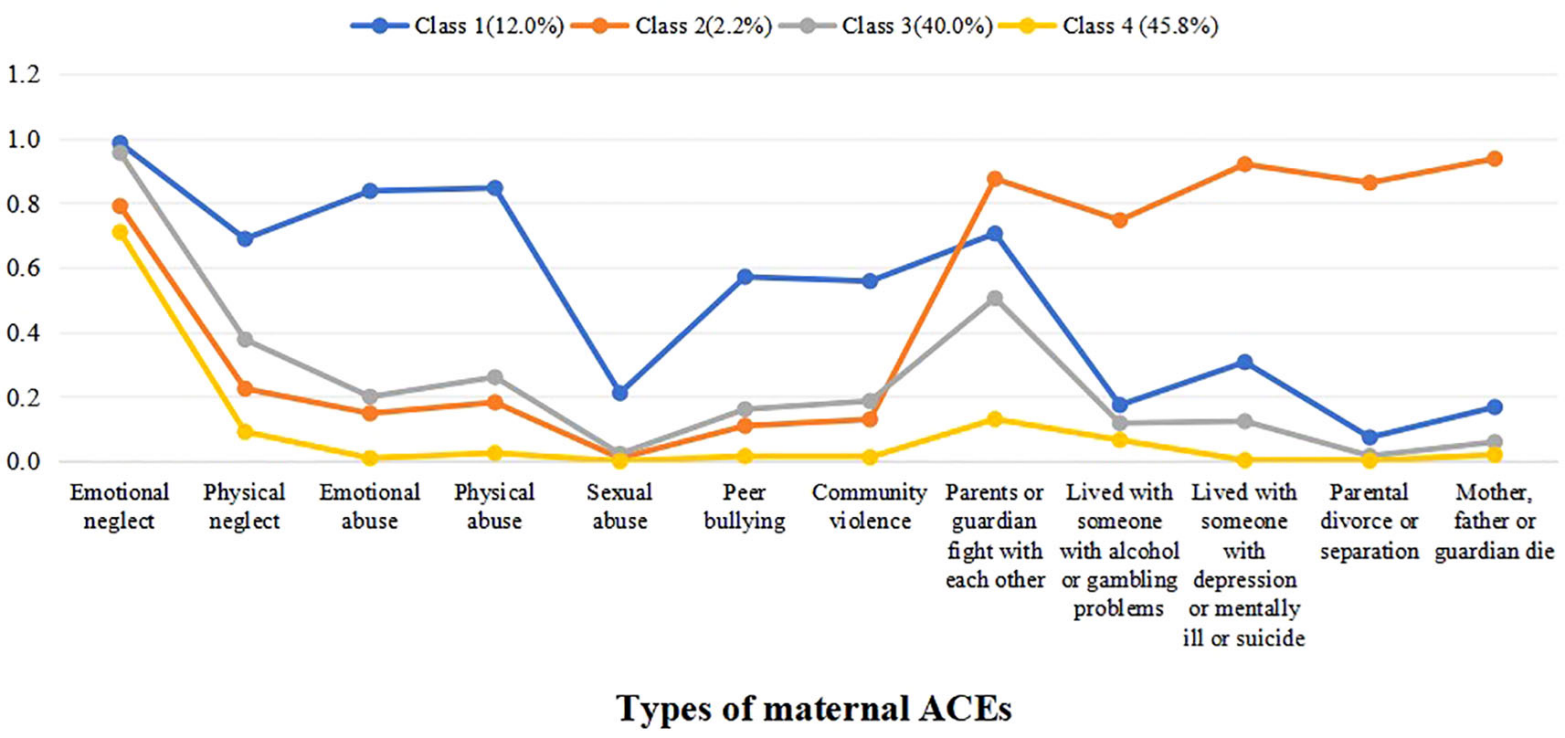
Plot of 4 latent classes of maternal adverse childhood experiences.

We examine the 4-class model of maternal ACEs and item-response probabilities for the 12 ACEs for each class (see **Figure 1**). The first class – high abuse and neglect (12.0%) – comprised high proportions of mothers who had childhood abuse and neglect experience. The second class – high household dysfunction (2.2%) – comprised high proportions of mothers likely to report household dysfunction in childhood. The third class – moderate ACEs (40.0%) – was characterized by mothers with medium proportions of overall ACEs in childhood. The fourth class – low ACEs (45.8%) – presented fairly low probability of exposure to each of the 12 ACEs in childhood.

### Association between types and patterns of maternal ACEs and preschool children’s EBPs

**Figure 2:**
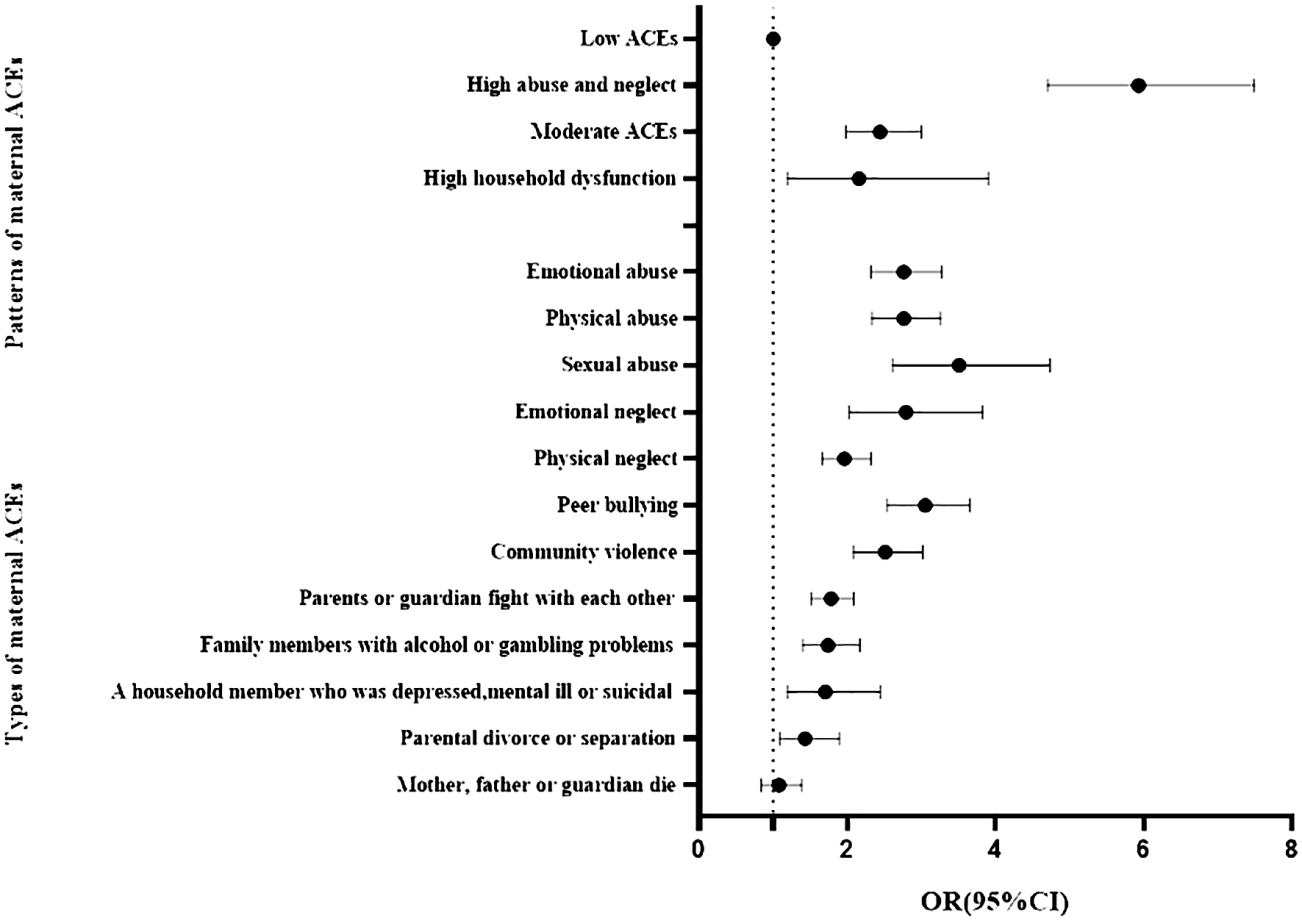
Logistic regression models of preschool children’s EBPs by types of maternal ACEs (Reference: 0 ACEs) and patterns of maternal ACEs (Reference: Low ACEs). Adjusted for residency, child gender, birth weight, maternal age, premature birth, maternal education level, family income, and family structure.

We examined the associations between types and patterns of maternal ACEs with children’s EBPs using binary logistic regression model. As shown in **Table 3**; **Figure 2**, when controlling for residency, child gender, birth weight, maternal age, premature birth, maternal education level, family income, and family structure, results showed that, compared with the low ACEs class, children of mothers in the high abuse and neglect class had the highest risk of developing EBPs (*OR* = 5.93, 95%*CI*: 4.70-7.49) in the total sample, followed by moderate ACEs class (*OR* = 2.44, 95%*CI*: 1.98-3.00), and high household dysfunction class (*OR* = 2.16, 95%*CI*: 1.19-3.90). Except for “Mother, father or guardian die”, other types of maternal ACEs all significantly related to children’s EBPs. The adjusted *OR* (95%*CI*) values ranged from 1.43 (95%*CI*: 1.09-1.89) to 3.51 (95%*CI*: 2.61-4.73).

**Table 3:** Number, percent, and odds ratio of preschool children EBPs from logistic regression models of types and patterns of maternal ACEs, n (%).

Maternal ACEs	n(%)	Emotional and behavioral problems	
		^a^ *OR*(95%*CI*)	*^b^OR*(95%*CI*)
Patterns			
Low ACEs	142(3.2)	1.0	1.0
High abuse and neglect	183(16.7)	6.04(4.80-7.61)^**^	5.93(4.70-7.49)^**^
Moderate ACEs	288(7.3)	2.39(1.95-2.94)^**^	2.44(1.98-3.00)^**^
High household dysfunction	13(6.7)	2.16(1.20-3.88)^*^	2.16(1.19-3.90)^*^
Types			
Emotional abuse	234(12.9)	2.82(2.37-3.34)^**^	2.76(2.32-3.28)^**^
Physical abuse	267(12.5)	2.85(2.42-3.37)^**^	2.76(2.33-3.26)^**^
Sexual abuse	61(18.2)	3.45(2.58-4.61)^**^	3.51(2.61-4.73)^**^
Emotional neglect	583(7.2)	2.65(1.93-3.63)^**^	2.79(2.03-3.83)^**^
Physical neglect	266(9.8)	2.00(1.69-2.36)^**^	1.96(1.66-2.32)^**^
Peer bullying	203(14.8)	3.22(2.69-3.85)^**^	3.05(2.54-3.65)^**^
Community violence	178(12.3)	2.43(2.02-2.92)^**^	2.51(2.08-3.02)^**^
Parents or guardian fight with each other	305(8.6)	1.71(1.45-2.01)	1.78(1.51-2.09)^**^
Family members with alcohol or gambling problems	108(10.4)	1.80(1.45-2.24)^**^	1.74(1.40-2.17)^**^
A household member who was depressed, mentally ill or suicidal	36(10.6)	1.74(1.22-2.49)^*^	1.70(1.19-2.44)^*^
Parental divorce or separation	64(9.0)	1.46(1.12-1.92)^*^	1.43(1.09-1.89)^*^
Mother, father or guardian die	77(7.0)	1.09(0.85-1.40)	1.08(0.84-1.38)

^a^: Unadjusted model; ^b^: Adjusted for residency, child gender, birth weight, maternal age, premature birth, maternal education level, family income, and family structure; ^**^: P<0.001; ^*^: P<0.05.

### Gender difference in the association between maternal ACEs and preschool children’s EBPs

**Figure 3:**
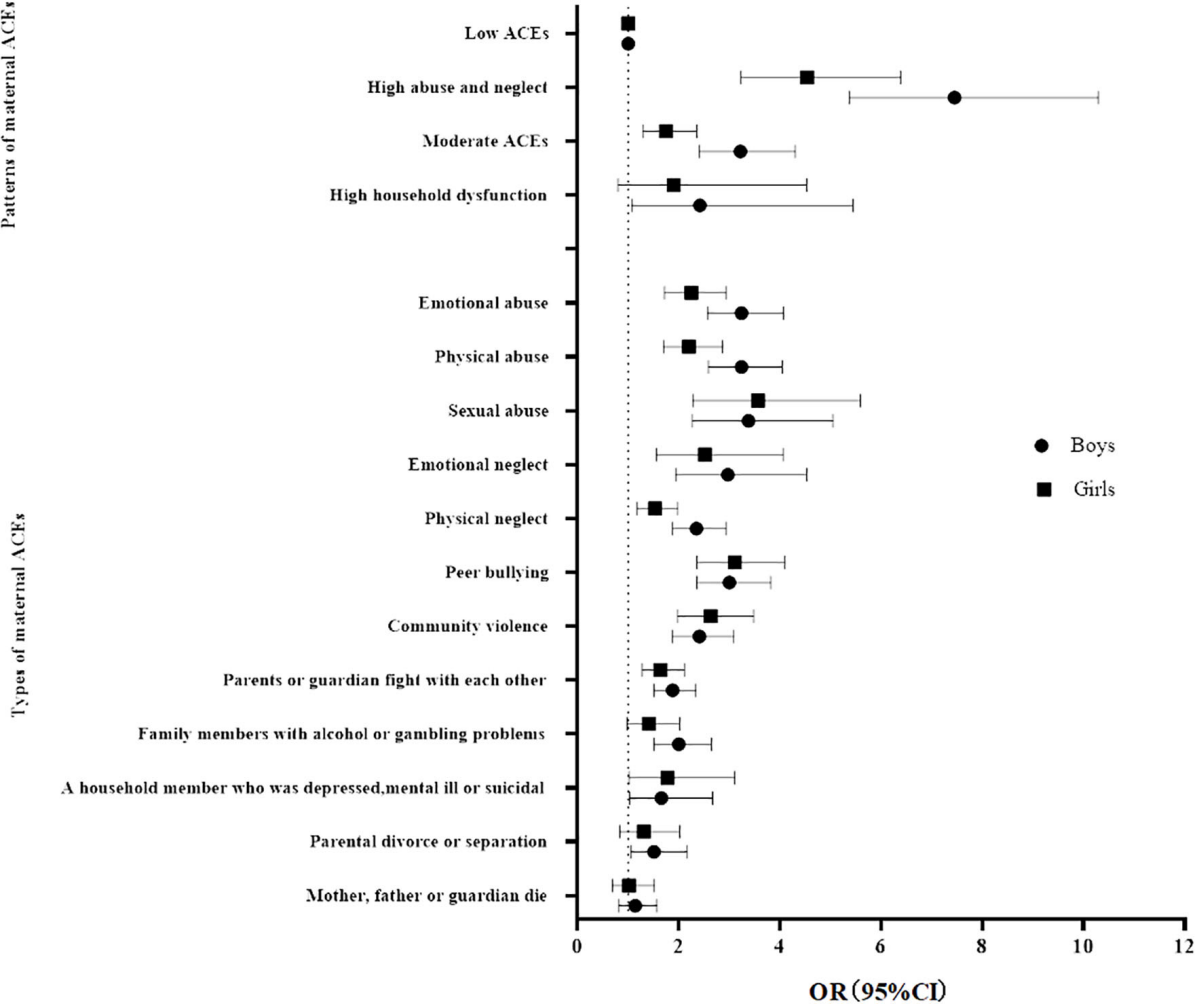
Logistic regression models of preschool children’s EBPs by types of maternal ACEs (Reference: 0 ACEs) and patterns of maternal ACEs (Reference: Low ACEs) in boys and girls, and the gender ratio. Adjusted for residency, child gender, birth weight, maternal age, premature birth, maternal education level, family income, and family structure.

As shown in **Figure 3**; **Supplementary Table S3**, when controlling for residency, birth weight, maternal age, premature birth, maternal education level, family income, and family structure, compared with the low ACEs class, the high abuse and neglect, moderate ACEs, and high household dysfunction classes of maternal ACEs were related to children’s EBPs, except that the high household dysfunction class was not related to children’s EBPs in girls (*OR*=1.90, 95%*CI*: 0.80-4.53). All types of maternal ACEs were related to children’s EBPs, except that “family members with alcohol or gambling problems”, “parental divorce or separation” and, “death of mother, father, or guardian” were not related to EBPs in girls. “death of mother, father, or guardian” was not associated with EBPs in boys.

Gender differences in high abuse and neglect class and moderate ACEs class were significant in relation to offspring’s EBPs (*ROR*=1.64, 95%*CI*:1.02-2.63; *ROR*=1.84, 95%*CI*:1.21-2.80). There were no gender difference in the influence of maternal ACEs types on children’s EBPs, except that the influence of maternal childhood physical abuse and physical neglect on children’s EBPs (*ROR*=1.47, 95%*CI*: 1.05-2.08; *ROR*=1.54, 95%*CI*: 1.09-2.16) was stronger in boys than girls.

### Sensitivity analysis

Due to the higher entropy of the 3-class group, we compared the results of different ACEs patterns grouping (**Supplementary Figure S2**). The results suggested that there was little difference in the association between exposure patterns of 3-class and 4-class maternal ACEs and children’s EBPs (**Supplementary Tables S4**, **S5**), indicating the robustness of the data.

In addition, we used different evaluation criteria of SDQ questionnaire to conduct sensitivity analysis on the data, and found that the sensitivity analysis results were basically consistent with this study. Results showed that 1680 preschoolers (17.4%) reported EBPs in the previous 6 months. Girls still had a higher risk of emotional symptoms than boys (*P*<0.001). Boys were still more likely to have hyperactivity, peer problems, prosocial behaviour problems and EBPs than girls (*P*<0.01) (**Supplementary Table S6**). Similarly, logistic regression analysis showed that compared with the low ACEs class, children of mothers in the high abuse and neglect class had the highest risk of developing EBPs (*OR* = 4.17, 95%*CI*: 3.56-4.88) in the total sample, followed by high household dysfunction class (*OR* = 2.00, 95%*CI*: 1.76-2.26) and moderate ACEs class (*OR* = 1.88, 95%*CI*: 1.29-2.73) (see **Supplementary Table S7**). Except for “Mother, father or guardian die”, other types of maternal ACEs all significantly related to children’s EBPs. The adjusted *OR* (95%CI) values ranged from 1.26 (95%*CI*: 1.04-1.52) to 2.90 (95%*CI*: 2.30-3.67). **Supplementary Table S8** showed that there was no gender difference in the influence of patterns and types of maternal ACEs on EBPs in preschool children. These findings indicated that the data and the results of different evaluation criteria for SDQ are robust.

## Discussion

We evaluated association between types and patterns of maternal ACEs and preschool children’s EBPs from a survey of preschools in China. We found that maternal childhood sexual abuse experience had the strongest correlation with children’s EBPs. Maternal high abuse and neglect class was most significantly related to offspring’s EBPs, followed by moderate ACEs class. Gender differences in maternal ACE exposure pattern (high abuse and neglect/moderate ACEs) were significant in relation to offspring’s EBPs.

In this study, the prevalence of maternal ACEs were higher than western countries (54, 55). The differences in the prevalence of maternal ACEs may be caused by various factors: the ACEs assessment questionnaire, different cultural background, different age groups and so on (8, 56, 57). The ACE-IQ questionnaire, employed in the present study, is more scientifically and rationally structured than other questionnaires, with a clear division into various dimensions and items. This enables a comprehensive accessment of ACEs, including dimensions of abuse, neglect, household dysfunction, and external violence (23). Furthermore, the questionnaire can be evaluated using either binary scoring or cumulative scoring. However, compared to other questionnaires, the ACE-IQ was only completed in 2016, resulting in a relatively limited number of studies utilizing it. In a scoping review of 49 studies examining the relationship between parental ACEs and children’s health, only two of these studies utilized the ACE-IQ questionnaire (58). It is anticipated that this questionnaire will be applied in more studies in the future, with a view to investigating the intergenerational effects of ACEs.

We found that maternal ACEs were associated with preschool children’s EBPs, which was consistent with other studies (59). Similarly, a cross-sectional study showed that maternal ACEs were related to higher depressive symptoms in offspring (60). These findings suggested that maternal ACEs were important factors affecting preschool children’s physical and mental health. In addition, as we known, ACEs include different types of abuse, neglect, household dysfunction, and so on. Prior studies found that different types of maternal ACEs may have different intergenerational effects on offspring. Pear VA et al. (61) demonstrated that maternal childhood physical abuse was significantly related to a 20% increased risk of child smoking, and that household alcohol abuse was associated with a possible 17% increased risk of tobacco use among children. A longitudinal study showed that compared with maternal childhood sexual abuse experience, maternal physical abuse experience is more significantly associated with the offspring’s internalising and externalising behaviour problems at the age of 12 (62). Chung et al. (63) found that, among the different domains of maternal ACEs (neglect, family distress, home violence and community violence), maternal home violence was associated with an increased risk of EBPs in children. Conversely, childhood neglect and family distress were observed to have a slight negative correlation with EBPs. A cross-sectional study showed that maternal community violence was associated with children’s externalizing problems, and that home violence was a risk factor for children’s internalizing problems (29). These studies indicate that different types of maternal ACEs have different effects on preschool children’s EBPs. At present, there are few studies on the correlations between different types of maternal ACEs and offspring’s EBPs in China. Relevant studies should be carried out in the future to provide reference for prevention and control of preschool children’s EBPs.

Previous studies focused on the impact of specific types of ACEs; however, in real life, different types of ACEs often occur simultaneously (64). Consequently, the LCA offered novel insights into the differential effects of maternal ACE patterns on offspring’s health. In current study, we classify maternal ACEs into four class: high abuse and neglect, high household dysfunction, moderate ACEs, and low ACEs, which is not entirely consistent with previous studies. For example, Ho et al. (23) identified three distinct ACE classes of Chinese young adults exposure patterns, which were labelled “low ACEs”, “emotionally and physically abused with intra-familial violence exposure”, and “multiple ACEs”. To examine the relationship between ACE exposure patterns and mental health, Elma et al. (65) identified three ACE exposure patterns, which were designated as “low risk”, “family maladjustment”, and “complex trauma exposure”. The discrepancies in the classification outcomes may be attributed to several factors. Firstly, the variation in the assessment approaches to ACEs (despite the utilization of the ACE-IQ questionnaire in both studies, the specific dimensions and items included differed) could influence the LCA classification of ACEs. Secondly, the classification results are also affected by factors such as age, gender, cultural background and socioeconomic level of the participants.

The present study sought to investigate the association between different patterns of maternal ACEs and offspring’s EBPs. The findings indicated that children of mothers with high abuse and neglect class demonstrated the highest risk of EBPs. Some scholars have explored the different exposure patterns of parental ACEs and the potential intergenerational health effects. For instance, a cohort study identified four classes of maternal ACEs exposure patterns and found that children whose mothers were in the category of “emotionally and physically abused with intra-familial violence exposure” were more likely to have low birth weight than those in other categories (66). Zhu et al. (67) identified a link between different classes of parental ACEs exposure patterns and children’s behavioral problems. Furthermore, they observed that the coparenting quality exerted a more pronounced moderating effect on the association between children’s behavioral problems and the “high ACEs” category than in the “low ACEs” category. These results indicate that children whose mothers have been subjected to high levels of abuse, neglect or violence are more prone to adverse health outcomes. This association can be explained from the perspective of attachment theory. Mothers who experience multiple types of early adversities, such as abuse, violence, and family members with mental illness, may develop psychological problems, increased parenting stress and exhibit negative parenting behaviors, all of which can affect parent-child interaction (68, 69). However, poor parent-child interaction may have a detrimental impact on children’s sense of security, impair self-regulation, and contribute to the mental development of mental health issues such as post-traumatic stress disorder (PTSD) (70, 71). Furthermore, research has indicated that mothers who have experienced high levels of abuse and violence, may exhibit dysfunctions of the prenatal hypothalamic-pituitary-adrenal (HPA) axis, which can impact the brain development of their offspring (72). Impaired brain development will lead to a series of psychological and behavioral problems during the growing process of children (73). The aforementioned studies indicate that assessment of maternal ACEs exposure patterns can help predict the combined risk of ACEs, thereby providing valuable information for the implementation of targeted preventive measures.

This study found that maternal childhood physical abuse/neglect had a greater impact on children’s EBPs in boys than girls, it indicated that there were gender differences in the association between maternal ACEs and children’s EBPs. This result can be explained by several studies. Past studies have found that mothers with ACEs are at higher risk for emotional problems (74, 75). Within the context of Chinese culture, mothers are the main caregivers of children, and their emotional problems have an important impact on offspring’s EBPs (40). Furthermore, research has demonstrated that boys are more likely to display a range of negative behaviors and emotional problems when interacting with mothers who have emotional problems (76). However, current studies on gender differences in the effects of maternal ACEs on offspring have yielded inconsistent. Roberts et al. (77) found that at the highest level of maternal childhood abuse, the risk of attention-deficit/hyperactivity disorder (ADHD) for female offspring was higher than that for male offspring, although the risk of ADHD for boys was also significantly increased compared to the children of mothers who have not experienced childhood abuse. In addition, a study found that maternal ACEs are related to children’s internalizing and externalizing problems and they found no gender differences in the relationship between maternal ACEs and children’s mental health (78). The inconsistency of these results may be related to the subjects (population, sample size, age, et al.), questionnaire used in the study, statistical methods and confounding factors controlled during the analysis (58, 79). At the same time, we found that maternal moderate ACEs class and high abuse and neglect class had a great influence on offspring’s EBPs in boys than girls. However, there are few studies on gender differences between maternal ACEs and offsprin’sg EBPs, and relevant conclusions need to be verified in more population researches.

## Strengths and limitations

A key strength of this study is to investigate the association of types and patterns of maternal ACEs with preschool children’s EBPs in China, and to examine gender differences related to these problems. The sample was large, which meant we could perform multivariate analysis, including gender differences.

However, several limitations are notable. Firstly, this is a cross-sectional study and we cannot infer a causal relationship between maternal ACEs and EBPs in preschool children. Nevertheless, our conclusions regarding the relevancy between maternal ACEs and offspring’s EBPs were in line with previous cohort researches (55, 80). Secondly, the ACEs questionnaire was retrospectively utilized to ask mothers about their exposure to any of the ACEs prior to turning 18. The retrospective nature of this research, which could not be avoided, may have created a risk of bias in the ACE survivors’ memories. Thirdly, there are many factors affecting preschool children’s emotional and behavioral problems, we should include more confounding factors in future studies. Finally, although this is a large sample study, the small number of mothers in the high household dysfunction class may have affected the stability of the results. It is recommended that cohort studies be conducted in the future to include a greater number of family and social environment factors that affect children’s EBPs in order to fully assess the impact of maternal ACEs exposure patterns on offspring’s EBPs. Furthermore, it is essential to further investigate the biological mechanism underlying the association between maternal ACEs and preschoolers’ EBPs in order to comprehend the intergenerational pathways of maternal ACEs.

## Conclusion

This study helps to better understand the relationship between maternal ACEs and EBPs in preschool children. This underscores the importance of early intervention to break the intergenerational impact of maternal ACEs. The study also suggested that using person-centred analytic methods can help to identify different classes of adversity experienced among mothers during childhood. Therefore, our study comprehensively considered the relationship between maternal ACE exposure patterns related to the risk of preschool children’s EBPs through identifying mothers exposed to the most problematic ACE patterns. These findings provide a chance to inform the promotion of the mental and physical health of children and adolescents.

## Implications

Our findings have significant implications for breaking the intergenerational transmission of maternal ACEs and implementing early intervention measures. At the policy level, it is necessary to reinforce legal protection for vulnerable groups such as women and children, to enhance penalties for violent behaviors, and to reduce the occurrence of ACEs (81). It is recommended that primary health care institutions and community workers conduct relevant knowledge seminars on ACEs, perform early screening for ACEs, identify at-risk families, and provide targeted intervention measures such as home visits, parental education training sessions, and mental health education seminars to enhance parents’ parenting skills (58, 82). It is incumbent upon educational institutions to provide a secure and supportive environment for students, with a particular focus on those who have been most severely affected by ACEs. This entails offspring psychological counseling, implementing measures to alleviate psychological distress, and taking steps to mitigate the adverse effects of ACEs (83). At the familial level, parent-child interaction therapy (PCIT) have been demonstrated to enhance parental self-regulation abilities and improve parent-child relationships. This approach has been shown to effectively reduce parental emotional and behavioral problems, as well as break the intergenerational transmission of ACEs (84, 85).

## Data Availability

The raw data supporting the conclusions of this article will be made available by the authors, without undue reservation.
