# Supplementary material for: Patterns of maternal adverse childhood experiences and the intergenerational association of preschool children’s emotional and behavioral problems
Source: Front Psychiatry. 2024 Aug 13;15:1431475. doi: 10.3389/fpsyt.2024.1431475 (PMC11347350; doi:10.3389/fpsyt.2024.1431475)
Supplement: Supplementary file 1 [file DataSheet1.docx]

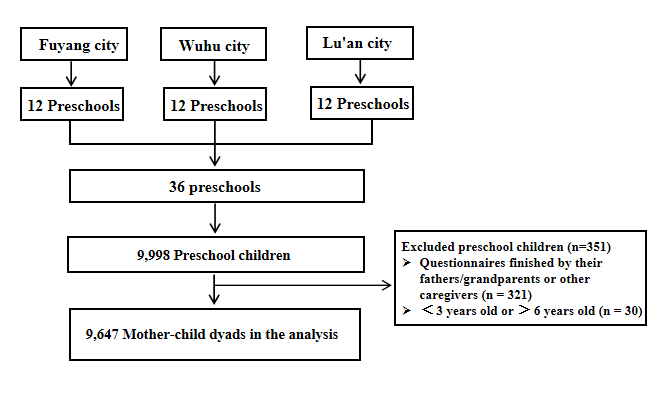


**Fig. S1 Flow chart.**


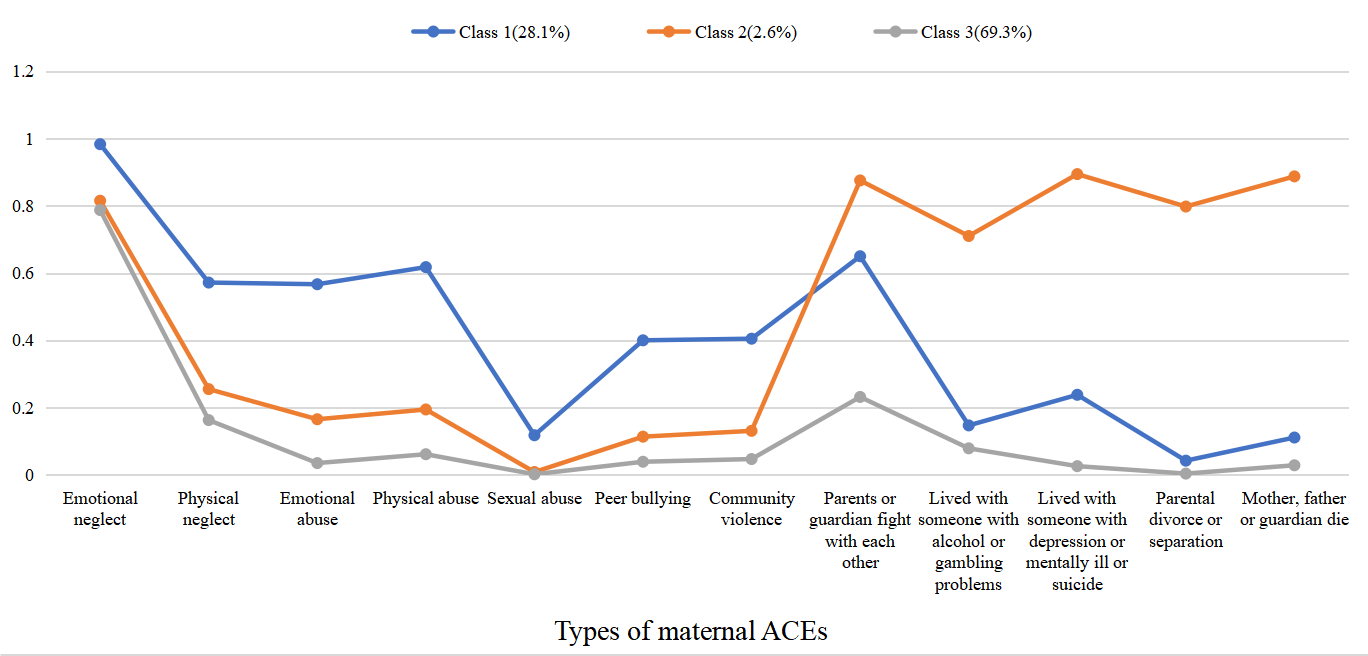


**Fig.S2 Plot of 3 latent classes of maternal adverse childhood experiences.**

**Tab.S1 The Strength and Difficulties Questionnaire (SDQ)**

| For each item, please mark the box for Not True, Somewhat True or Certainly True. Please give your answers on the basis of the child's behaviour over the last six months or this school year. | |
| --- | --- |
| **EBPs** | **Question** |
| 1 | Considerate of other people's feelings |
| 2 | Restless, overactive, cannot stay still for long |
| 3 | Often complains of headaches, stomach-aches or sickness |
| 4 | Shares readily with other children (treats, toys, pencils etc) |
| 5 | Often has temper tantrums or hot tempers |
| 6 | Rather solitary, tends to play alone |
| 7 | Generally obedient, usually does what adults request |
| 8 | Many worries, often seems worried |
| 9 | Helpful if someone is hurt, upset or feeling ill |
| 10 | Constantly fidgeting or squirming |
| 11 | Has at least one good friend |
| 12 | Often fights with other children or bullies them |
| 13 | Often unhappy, down-hearted or tearful |
| 14 | Generally liked by other children |
| 15 | Easily distracted, concentration wanders |
| 16 | Nervous or clingy in new situations, easily loses confidence |
| 17 | Kind to younger children |
| 18 | Often lies or cheats |
| 19 | Picked on or bullied by other children |
| 20 | Often volunteers to help others (parents, teachers, other children) |
| 21 | Thinks things out before acting |
| 22 | Steals from home, school or elsewhere |
| 23 | Gets on better with adults than with other children |
| 24 | Many fears, easily scared |
| 25 | Sees tasks through to the end, good attention span |

**Tab.S2 Prevalence of each type of maternal adverse childhood experiences (N = 9,647).**

| Maternal ACEs |  | Numbers | Prevalence(%) |
| --- | --- | --- | --- |
| **Type of ACEs** |  |  |  |
| Emotional abuse | Yes | 1812 | 18.8 |
| Physical abuse | Yes | 2133 | 22.1 |
| Sexual abuse | Yes | 335 | 3.5 |
| Emotional neglect | Yes | 8130 | 84.3 |
| Physical neglect | Yes | 2702 | 28.0 |
| Peer bullying | Yes | 1374 | 14.2 |
| Community violence | Yes | 1445 | 15.0 |
| Parents or guardian fight with each other | Yes | 3529 | 36.6 |
| Family members with alcohol or  gambling problems | Yes | 1043 | 10.8 |
| A household member who was depressed, mentally ill or suicidal | Yes | 341 | 3.5 |
| Parental divorce or separation | Yes | 715 | 7.4 |
| Mother, father or guardian die | Yes | 1105 | 11.5 |
| **Number of ACEs type** |  |  |  |
| 0 |  | 954 | 9.9 |
| 1 |  | 2686 | 27.8 |
| 2 |  | 2081 | 21.6 |
| 3 |  | 1373 | 14.2 |
| 4+ |  | 2553 | 26.5 |

**Tab.S3 Number, percent and odds ratio for preschool children’s EBPs from logistic regression models of maternal ACEs in girls and boys, n(%)**

| Maternal ACEs | Boys | | |  | Girls | | |  | Ratio of two odds ratios in boys versus girls^c^ | |
| --- | --- | --- | --- | --- | --- | --- | --- | --- | --- | --- |
|  | n(%) | *OR*(95%*CI*)^a^ | *P*-value |  | n(%) | *OR*(95%*CI*)^a^ | *P*-value |  | Ratio of two odds ratios(*ROR)* | *P*-value |
| **Patterns** |  |  |  |  |  |  |  |  |  |  |
| Low ACEs | 68(3.0) | 1.0 |  |  | 74(3.5) | 1.0 |  |  |  |  |
| High abuse and neglect | 106(18.3) | 7.45(5.38-10.29) | ＜0.001 |  | 77(14.9) | 4.54(3.23-6.39) | ＜0.001 |  | 1.64(1.02-2.63) | 0.039 |
| Moderate ACEs | 174(8.7) | 3.22(2.41-4.30) | ＜0.001 |  | 114(5.9) | 1.75(1.29-2.36) | ＜0.001 |  | 1.84(1.21-2.80) | 0.004 |
| High household dysfunction | 7(6.7) | 2.42(1.07-5.45) | 0.033 |  | 6(6.6) | 1.90(0.80-4.53) | 0.147 |  | 1.27(0.39-4.18) | 0.690 |
| **Types** |  |  |  |  |  |  |  |  |  |  |
| Emotional abuse | 142(15.4) | 3.24(2.57-4.07) | ＜0.001 |  | 92(10.4) | 2.25(1.72-2.93) | ＜0.001 |  | 1.44(1.00-2.05) | 0.050 |
| Physical abuse | 164(14.7) | 3.24(2.59-4.05) | ＜0.001 |  | 103(10.1) | 2.20(1.70-2.86) | ＜0.001 |  | 1.47(1.05-2.08) | 0.027 |
| Sexual abuse | 34(18.9) | 3.38(2.27-5.05) | ＜0.001 |  | 27(17.4) | 3.57(2.28-5.59) | ＜0.001 |  | 0.95(0.52-1.73) | 0.858 |
| Emotional neglect | 331(7.9) | 2.97(1.94-4.53) | ＜0.001 |  | 252(6.4) | 2.52(1.56-4.06) | ＜0.001 |  | 1.18(0.62-2.23) | 0.614 |
| Physical neglect | 165(11.7) | 2.35(1.88-2.93) | ＜0.001 |  | 101(7.8) | 1.53(1.18-1.98) | 0.001 |  | 1.54(1.09-2.16) | 0.014 |
| Peer bullying | 114(15.7) | 3.00(2.36-3.82) | ＜0.001 |  | 89(13.8) | 3.11(2.36-4.09) | ＜0.001 |  | 0.96(0.67-1.39) | 0.847 |
| Community violence | 99(13.0) | 2.41(1.88-3.09) | ＜0.001 |  | 79(11.5) | 2.63(1.98-3.48) | ＜0.001 |  | 0.92(0.63-1.33) | 0.649 |
| Parents or guardian fight with each other | 177(9.7) | 1.88(1.51-2.34) | ＜0.001 |  | 128(7.5) | 1.64(1.28-2.11) | ＜0.001 |  | 1.15(0.82-1.60) | 0.421 |
| Family members with alcohol or gambling problems | 70(12.2) | 2.00(1.51-2.65) | ＜0.001 |  | 38(8.1) | 1.41(0.98-2.02) | 0.065 |  | 1.42(0.90-2.24) | 0.135 |
| A household member who was depressed, mentally ill or suicidal | 21(11.1) | 1.66(1.03-2.67) | 0.036 |  | 15(9.9) | 1.78(1.02-3.11) | 0.042 |  | 0.93(0.45-1.94) | 0.852 |
| Parental divorce or separation | 39(10.2) | 1.51(1.06-2.16) | 0.023 |  | 25(7.6) | 1.31(0.84-2.02) | 0.232 |  | 1.15(0.66-2.03) | 0.622 |
| Mother, father or guardian die | 47(8.0) | 1.14(0.82-1.57) | 0.444 |  | 30(5.8) | 1.02(0.69-1.52) | 0.927 |  | 1.12(0.67-1.86) | 0.670 |

^a^: Adjusted for residency, birth weight, maternal age, premature birth, maternal education level, family income, and family structure; c: Calculated by adjusted odds ratio.

**Tab.S4 Number, percent, and odds ratio of preschool children’s EBPs from logistic regression models of maternal ACEs, n (%).**

| Maternal ACEs | Emotional and behavioral problems | | |
| --- | --- | --- | --- |
|  | n(%) | *OR*(95%*CI*)^a^ | *OR*(95%*CI*)^b^ |
| **Patterns** |  |  |  |
| Low ACEs | 6834(70.8) | 1.0 | 1.0 |
| High abuse and neglect | 2581(26.8) | 3.37(2.85-3.98)^**^ | 3.31(2.80-3.91)^**^ |
| High household dysfunction | 232(2.4) | 1.84(1.11-3.05)^*^ | 1.82(1.09-3.04)^*^ |

^a^: Unadjusted model; ^b^: Adjusted for residency, child gender, birth weight, maternal age, premature birth, maternal education level, family income, and family structure; ^**^: *P*＜0.001; ^*^: *P*＜0.05.

**Tab.S5 Number, percent and odds ratio for preschool children’s EBPs from logistic regression models of maternal ACEs in girls and boys, n(%)**

| Maternal ACEs | Boys | | |  | Girls | | |  | Ratio of two odds ratios in boys versus girls^c^ | |
| --- | --- | --- | --- | --- | --- | --- | --- | --- | --- | --- |
|  | n(%) | *OR*(95%*CI*)^a^ | *P*-value |  | n(%) | *OR*(95%*CI*)^a^ | *P*-value |  | Ratio of two odds ratios(*ROR)* | *P*-value |
| **Patterns** |  |  |  |  |  |  |  |  |  |  |
| Low ACEs | 3512(70.5) | 1.0 |  |  | 3322(71.2) | 1.0 |  |  | 1.0 |  |
| High abuse and neglect | 1343(27.0) | 3.94(3.14-4.94) | ＜0.001 |  | 1238(26.5) | 2.66(2.06-3.43) | ＜0.001 |  | 1.48(1.05-2.08) | 0.024 |
| High household dysfunction | 128(2.6) | 1.98(1.01-3.88) | 0.047 |  | 104(2.2) | 1.65(0.74-3.65) | 0.219 |  | 1.20(0.42-3.41) | 0.732 |

^a^: Adjusted for residency, birth weight, maternal age, premature birth, maternal education level, family income, and family structure.

**Tab. S6 Background and demographic information for overall sample**

| Variables |  | Total | Boys(N,%) | Girls(N,%) | *P*-value |
| --- | --- | --- | --- | --- | --- |
| Emotional Problems | Normal | 8077(83.7) | 4254(52.7) | 3823(47.3) | ＜0.001 |
|  | Abnormal | 1570(16.3) | 729(46.4) | 841(53.6) |  |
| Conduct Problems | Normal | 7884(81.7) | 4028(51.1) | 3856(48.9) | 0.019 |
|  | Abnormal | 1763(18.3) | 955(54.2) | 808(45.8) |  |
| Hyperactivity-Inattention | Normal | 7453(77.3) | 3684(49.4) | 3769(50.6) | ＜0.001 |
|  | Abnormal | 2194(22.7) | 1299(59.2) | 895(40.8) |  |
| Peer Problems | Normal | 6348(65.8) | 3157(49.7) | 3191(50.3) | ＜0.001 |
|  | Abnormal | 3299(34.2) | 1826(55.4) | 1473(44.6) |  |
| Prosocial Problems | Normal | 7863(81.5) | 3925(49.9) | 3938(40.1) | ＜0.001 |
|  | Abnormal | 1784(18.5) | 1058(59.3) | 726(40.7) |  |
| EBPs | Normal | 7967(82.6) | 4045(50.8) | 3922(49.2) | ＜0.001 |
|  | Abnormal | 1680(17.4) | 938(55.8) | 742(44.2) |  |

**Tab.S7 Number, percent, and odds ratio of** **preschool children’s EBPs from logistic regression models of maternal ACEs, n (%).**

| Maternal ACEs | Emotional and behavioral problems | | |
| --- | --- | --- | --- |
|  | n(%) | *OR*(95%*CI*)^a^ | *OR*(95%*CI*)^b^ |
| **Patterns** |  |  |  |
| Low ACEs | 494(11.1) | 1.0 | 1.0 |
| High abuse and neglect | 376(34.2) | 4.15(3.55-4.85)^**^ | 4.17(3.56-4.88)^**^ |
| Moderate ACEs | 773(19.7) | 1.96(1.73-2.21)^**^ | 2.00(1.76-2.26)^**^ |
| High household dysfunction | 37(19.0) | 1.87(1.29-2.70)^*^ | 1.88(1.29-2.73)^*^ |
| **Types** |  |  |  |
| Emotional abuse | 535(29.5) | 2.45(2.17-2.76)^**^ | 2.44(2.16-2.76)^**^ |
| Physical abuse | 600(28.1) | 2.33(2.08-2.61)^**^ | 2.29(2.04-2.57)^**^ |
| Sexual abuse | 121(36.1) | 2.81(2.24-3.54)^**^ | 2.90(2.30-3.67)^**^ |
| Emotional neglect | 1558(19.2) | 2.71(2.24-3.29)^**^ | 2.86(2.35-3.48)^**^ |
| Physical neglect | 648(24.0) | 1.81(1.62-2.02)^**^ | 1.80(1.61-2.01)^**^ |
| Peer bullying | 440(32.0) | 2.67(2.35-3.04)^**^ | 2.59(2.27-2.95)^**^ |
| Community violence | 388(26.9) | 1.96(1.72-2.24)^**^ | 2.03(1.78-2.32)^**^ |
| Parents or guardian fight with each other | 756(21.4) | 1.53(1.38-1.71)^**^ | 1.59(1.42-1.77)^**^ |
| Family members with alcohol or gambling problems | 251(24.1) | 1.59(1.37-1.85)^**^ | 1.55(1.33-1.81)^**^ |
| A household member who was depressed, mentally ill or suicidal | 91(26.7) | 1.77(1.38-2.26)^**^ | 1.77(1.37-2.27)^**^ |
| Parental divorce or separation | 150(20.0) | 1.28(1.06-1.55)^*^ | 1.26(1.04-1.52)^*^ |
| Mother, father or guardian die | 205(18.6) | 1.09(0.93-1.28) | 1.09(0.93-1.29) |

^a^: Unadjusted model; ^b^: Adjusted for residency, child gender, birth weight, maternal age, premature birth, maternal education level, family income, and family structure; ^**^: *P*＜0.001; ^*^: *P*＜0.05.

**Tab.S8 Number, percent and odds ratio for preschool children’s EBPs from logistic regression models of maternal ACEs in girls and boys, n(%)**

| Maternal ACEs | Boys | | |  | Girls | | |  | Ratio of two odds ratios in boys versus girls^c^ | |
| --- | --- | --- | --- | --- | --- | --- | --- | --- | --- | --- |
|  | n(%) | *OR*(95%*CI*)^a^ | *P*-value |  | n(%) | *OR*(95%*CI*)^a^ | *P*-value |  | Ratio of two odds  ratios (*ROR*) | *P*-value |
| **Patterns** |  |  |  |  |  |  |  |  |  |  |
| Low ACEs | 269(11.7) | 1.0 |  |  | 225(10.5) | 1.0 |  |  | 1.0 |  |
| High abuse and neglect | 213(36.7) | 4.51(3.63-5.59) | ＜0.001 |  | 163(31.5) | 3.76(2.97-4.76) | ＜0.001 |  | 1.20(0.87-1.65) | 0.265 |
| Moderate ACEs | 437(21.9) | 2.17(1.83-2.56) | ＜0.001 |  | 336(17.5) | 1.80(1.50-2.16) | ＜0.001 |  | 1.21(0.94-1.54) | 0.139 |
| High household dysfunction | 19(18.3) | 1.74(1.03-2.93) | 0.037 |  | 18(19.8) | 2.04(1.19-3.50) | 0.010 |  | 0.85(0.40-1.81) | 0.678 |
| **Types** |  |  |  |  |  |  |  |  |  |  |
| Emotional abuse | 308(33.3) | 2.73(2.31-3.21) | ＜0.001 |  | 227(25.6) | 2.14(1.79-2.56) | ＜0.001 |  | 1.28(1.00-1.63) | 0.050 |
| Physical abuse | 347(31.1) | 2.48(2.12-2.90) | ＜0.001 |  | 253(24.8) | 2.07(1.74-2.46) | ＜0.001 |  | 1.20(0.95-1.51) | 0.129 |
| Sexual abuse | 66(36.7) | 2.77(2.01-3.81) | ＜0.001 |  | 55(35.5) | 3.03(2.14-4.29) | ＜0.001 |  | 0.91(0.57-1.47) | 0.710 |
| Emotional neglect | 864(20.7) | 2.75(2.13-3.54) | ＜0.001 |  | 694(17.5) | 3.01(2.21-4.09) | ＜0.001 |  | 0.91(0.61-1.36) | 0.657 |
| Physical neglect | 367(26.0) | 1.85(1.59-2.15) | ＜0.001 |  | 281(21.7) | 1.74(1.47-2.05) | ＜0.001 |  | 1.06(0.85-1.33) | 0.593 |
| Peer bullying | 255(35.1) | 2.77(2.32-3.30) | ＜0.001 |  | 185(28.6) | 2.37(1.94-2.88) | ＜0.001 |  | 1.17(0.90-1.52) | 0.248 |
| Community violence | 218(28.7) | 2.05(1.72-2.46) | ＜0.001 |  | 170(24.8) | 2.00(1.64-2.44) | ＜0.001 |  | 1.03(0.78-1.34) | 0.856 |
| Parents or guardian fight with each other | 433(23.7) | 1.71(1.47-1.98) | ＜0.001 |  | 323(19.0) | 1.45(1.23-1.70) | ＜0.001 |  | 1.18(0.95-1.47) | 0.142 |
| Family members with alcohol or gambling problems | 140(24.5) | 1.46(1.19-1.80) | ＜0.001 |  | 111(23.6) | 1.67(1.32-2.11) | ＜0.001 |  | 0.87(0.64-1.20) | 0.400 |
| A household member who was depressed, mentally ill or suicidal | 52(27.5) | 1.70(1.22-2.37) | 0.002 |  | 39(25.7) | 1.85(1.27-2.71) | 0.002 |  | 0.92(0.56-1.52) | 0.742 |
| Parental divorce or separation | 81(21.1) | 1.17(0.90-1.51) | 0.253 |  | 69(20.8) | 1.37(1.03-1.82) | 0.029 |  | 0.85(0.58-1.25) | 0.421 |
| Mother, father or guardian die | 112(19.0) | 1.01(0.81-1.27) | 0.918 |  | 93(18.0) | 1.20(0.94-1.54) | 0.137 |  | 0.84(0.60-1.18) | 0.312 |

^a^: Adjusted for residency, birth weight, maternal age, premature birth, maternal education level, family income, and family structure; ^c^: Calculated by adjusted odds ratio.
